# Creating change through leadership development: an overview of the 2019-2021 Canadian Health Libraries Leadership Institute

**DOI:** 10.29173/jchla29755

**Published:** 2024-04-01

**Authors:** Sandy Iverson, Miriam Ticoll

**Affiliations:** 1 CHLA/ABSC President, 2019-2020; 2 CHLA/ABSC President, 2011-2012

In 2019, the Canadian Health Libraries Association/Association des bibliothèques de la santé du Canada (CHLA/ABSC) launched the first Leadership Institute for health librarians in Canada. This article provides an overview of how the Leadership Institute was developed and delivered, the content and structure of the Leadership Institute, lessons learned from evaluations of the program, and reflections of participants two years after the program was launched.

## Why a Leadership Institute for health librarians?

The need for a Leadership Institute was driven by several factors affecting health science libraries, librarians, and information specialists:

- A rapidly changing health science and health information environment – including advances in artificial intelligence and personalized medicine – necessitating creative vision and energetic engagement of health science librarians and information specialists.
- A leadership gap because of baby-boomer retirements.
- A trend in academic health libraries to fill leadership positions from outside of Canada and rarely by people from visible minorities, as well as a disproportionate number of men in leadership positions despite the preponderance of women in the field.
- Under-resourced hospital libraries providing few leadership growth opportunities for their staff. With the merging of hospitals into larger health networks, librarians struggle to position themselves to be of obvious service in these newly structured organizations. Without adequate leadership to advocate for their important role in evidence-based decision making, library positions may disappear entirely.
- An imperative for health librarians to be leaders in the vanguard of innovation – professionals who are comfortable with and capable of measuring and articulating the value of library services.

These trends also have implications for the long-term viability of professional associations such as CHLA/ABSC.

## Planning and designing the Leadership Institute

In the summer of 2017, the Association’s Board of Directors formed a Leadership Institute steering committee^2^. During the following year the steering committee undertook several consultation and planning activities culminating in a formal proposal to the Board.

The steering committee explored programs of other leadership institutes for librarians, including the Canadian Northern Exposure to Leadership Institute and the United States-based Leadership Fellows Program sponsored by the Association of Academic Health Sciences Libraries and the National Library of Medicine [1]. The committee also informally discussed the idea with a variety of library leaders for input. In addition, a survey of CHLA/ABSC members and an in-person focus group were critical to help shape the content and format of the Institute and the development of a detailed proposal for the CHLA/ABSC Board (see Online Supplement, Appendix 1).

Supplement Appendix 1

In the winter of 2018, the steering committee surveyed the CHLA/ABSC membership to gauge interest and collect feedback on some preliminary ideas. 64 members out of a total membership of 310 responded. Respondents generally believed this idea would benefit the profession and were generous about sharing their ideas and opinions, providing many comments and suggestions. Concerns were expressed about the potential fees to participate and the need to receive value for investment.

In June of 2018, at CHLA/ABSC’s annual conference, the steering committee invited Association members to attend a focus group to further explore what people would like to see in a Leadership Institute. 15 members (plus facilitators) attended the focus group. The steering committee shared the results of the survey with the participants and then asked for their input to help guide the development of the curriculum and the structure of the Institute.

In the member survey, the steering committee proposed three broad themes for curriculum content:

- Leadership & Management Skills
- Emerging Library Trends
- Developments in Healthcare

The committee asked the focus group participants if these thematic areas and proposed related subtopics accurately reflected the primary interests within health librarianship more broadly, and to brainstorm additional themes and subtopics. Of the proposed themes, Leadership & Management Skills was perceived as having the most value by the focus group members, Emerging Trends in Library Science had some interest, and Developments in Healthcare was perceived to have the least value.

Various proposed subtopics in Leadership & Management received a fair amount of support but new topics added by the focus group participants received even more support. These included:

- Relationship-building
- Collaboration
- Change Management
- Advocacy

The focus group members were overwhelmingly interested in seeing the Leadership Institute focus on strategic thinking and strategic planning. The following year Sandy Iverson delivered a workshop on leadership development at the 2019 European Association for Health Information and Libraries (EAHIL) conference [2], where she facilitated a repeat of the same exercise with about 30 health librarians from around the world. The results were the same an overwhelming desire to see any Leadership Institute for health librarians focus on strategic thinking and strategic planning.

Incorporating the findings of the survey and focus group as well as research it had conducted, the steering committee felt confident submitting a detailed formal proposal to the CHLA/ABSC Board of Directors, which was approved at the Fall 2018 Board meeting.

## Program structure, content, and delivery

Following the approval of the proposal, the steering committee began detailed planning of the components of the Leadership Institute and launched a call for participants (see Online Supplement, Appendix 2). The call for Leadership Institute participant applications was distributed by the CHLA/ABSC communications channels. Applicants had to be graduates from an accredited library and information science program, have a minimum of four years post-graduate work experience, and have worked at least two years in a health environment. Applicants were invited to submit a cover letter, a letter from their employer or other industry supporter, and a resume. Applications were reviewed by the steering committee. 12 applicants were selected.

Supplement Appendix 2

The four main components of the Leadership Institute were as follows:

- A 1.5-day in-person intensive retreat to kick off the program, introducing the framework, setting goals and expectations and facilitation of connections between participants and mentors.
- Eight virtual learning and discussion sessions held throughout the year, including presentations by leading practitioners in health or information services, followed by group discussions and exercises.
- A project (known as a “capstone project”) designed and delivered by each of the participants in which they were expected to apply and demonstrate what they were learning during the program.
- A closing in-person event, planned as a full day retreat to take place in June, 2020, was an opportunity for participants to reflect on what they had learned and to present their capstone projects and receive structured constructive feedback. The Covid pandemic forced the Leadership Institute to extend an additional 10 months as participants struggled to cope with pandemic related challenges. The capstone project presentations were delivered virtually instead of in-person as had been originally planned.

All steering committee members and 2 additional CHLA/ABSC members^3^ were recruited to act as mentors and any participant could reach out to any mentor during the program for feedback, guidance, or simply have a chat. Participants were invited to discuss topics covered in the sessions, their capstone projects, career opportunities or any other professional questions or concerns.

Both the beginning and concluding residential components were scheduled at times and in locations adjacent to the Association’s annual conference. CHLA/ABSC engaged Rebecca Jones, a well-known library consultant and facilitator with experience in delivering leadership content, including strategic planning and strategic thinking to librarians in other sectors. The steering committee worked with her to develop and deliver the final curriculum for the Leadership Institute (see Online Supplement, Appendix 3). A library school student on a practicum project developed an online portal where participants could access resources and share information. Elsevier and Wolters Kluwer both contributed industry support funding (totaling over $10,000). Elsevier also contributed the expertise of Jean Shipman, their library ambassador at the time and an experienced leader in health libraries in the United States. Ms. Shipman delivered guest lectures and generously shared her extensive experience with Leadership Institute participants at the opening retreat.

Supplement Appendix 3

The Leadership Institute’s core curriculum drew on two leadership frameworks:

1. The Five Mind Sets for Management [3] developed by H. Mintzberg, a renowned management researcher and professor at McGill University and Harvard University, along with his colleague J. Gosling.
2. The LEADS Framework from the Canadian College of Health Leaders/Collège canadiens des leaders en santé [4].

The over-arching goal of the Leadership Institute was to provide an opportunity for participants to develop a leadership approach that integrates the five critical management mindsets identified by Mintzberg & Gosling: (*i*) managing self, (*ii*) managing organizations, (*iii*) managing context, (*iv*) managing relationships, and (*v*) managing change. These aligned well with the LEADS Framework which presents a common understanding of what good leadership looks, feels, and sounds like across all levels of service provision in healthcare.

During the initial intensive, participants were exposed to these frameworks through self-reflection exercises (see Online Supplement, Appendix 4), small and large group work, and presentations by the facilitator and mentors about their real-world experiences as leaders in health libraries.

Supplement Appendix 4

## Evaluation

As part of her contract, Rebecca Jones was charged with producing an evaluative report of the Leadership Institute. She asked all participants to complete an evaluation form (see Online Supplement, Appendix 5) and to write a reflective essay, and she submitted an evaluative report based on the evaluations she received from participants. Nine of the 12 participants completed the evaluation. These are highlights from the evaluation:

- Almost all content was rated as contributing to growth in all areas for almost all participants.
- In all areas of learning participants indicated that they benefited from an intentional, focused curriculum that allowed them to step away from their daily tasks and build awareness of leadership issues tailored to their sector by engaging with mentors, peers, and the material presented.
- All respondents were able to identify at least two tools that they are now comfortable using which were not previously at their disposal.
- >75% of respondents indicated they “strongly agree” that they now have a network of peers and mentors to turn to, while the remaining ~25% said they “somewhat agree.”
- >75% of respondents indicated they “strongly agree” that they would recommend the Institute to other information professionals or librarians in the health sector, while the remaining ~25% said they “Somewhat agree.”
- Based on participants qualitative comments, the most valued components of the Leadership Institute were:
  - starting the cohort with the residential intensive;
  - the interaction with mentors from the field;
  - curriculum on diversity, equity, and inclusion

Supplement Appendix 5

## Did the Leadership Institute foster more leadership in participants?

The steering committee was pleased with the feedback and felt confident it had delivered a program that the participants enjoyed. But to what degree had the Leadership Institute produced leaders? Did participants perceive themselves to be more prepared to assume professional roles with more leadership responsibilities since the start of the Leadership Institute? To answer these questions, early in 2023, almost two years after the official ending of the Leadership Institute, two members of the steering committee, the authors of this paper, conducted individual semi-structured video-conference interviews with each of the twelve Leadership Institute participants.

The findings were as follows:

- 100% of participants would still recommend the program to colleagues.
- 7/12 participants have assumed (either permanently or temporarily) professional roles with more leadership responsibilities since the launch of the LI in 2019.
- Benefits mentioned by several of the participants included: increased confidence, a better understanding of the value of strategic alignment, and an ongoing use of the mindset framework to help them understand or analyze a variety of professional situations.
- Most participants noted increased involvement in committee work either in their workplaces or volunteer commitments, including professional associations. 50% of the participants served on the board of CHLA/ABSC either during their involvement with the LI or after the program.
- Most participants commented on the value of now having a professional cohort or network of people across the country with whom they can work or draw support - including mentors and other participants.

## Lessons for future Leadership Institutes

Several participants interviewed indicated that one of the aspects of the program that they appreciated was what we might term “credentialism.” Participation in the Leadership Institute communicated to their employers, potential employers, and advancement committees, that they were not only interested in assuming leadership roles, but they had taken steps to gain additional expertise by enrolling in a leadership program.

The mentorship program was mentioned positively by most participants who were interviewed. They appreciated the mentors and found their involvement in the Leadership Institute very valuable. While some people really enjoyed the unstructured nature of the program with respect to mentorship –participants choosing to reach out to as many or as few of the mentors as they wished –others felt they would have benefitted more by having an assigned mentor. This suggests that there was a lack of clarity in how the mentoring was supposed to work. This could be improved upon by highlighting each mentor’s skills and interests and by asking mentors to be more proactive in reaching out to participants and participating in more of the group activities such as the online sessions.

Another aspect of the Leadership Institute that most participants commented on was the opening 1.5-day in- person retreat. They found this to be the most rewarding aspect of the program and wished that (*i*) it had been longer and (*ii*) that the closing face-to-face retreat had been able to go ahead. Virtual networking and learning have their benefits, but the opportunity to meet in a retreat setting, with limited distractions proved to be highly valued by all participants.

The program tried as much as possible to emphasize that leadership can be practiced no matter what one’s position is in an organization. Leadership is having ideas and constructive opinions and the confidence to communicate them effectively, whether in a staff meeting, a family, or at a professional conference. It is stepping up to lead a discussion, organize a party, or take notes. Leadership is the ability to lead, influence, or guide other people, teams, or organizations. Nonetheless a few participants struggled with the difference between being a leader and being in a management position and indicated that they would have liked a few more “practical” or managerial skills development opportunities.

## Conclusion

All 12 Leadership Institute participants came away from their experience with an increased understanding of leadership as well as increased confidence in assuming leadership and would recommend this program to their colleagues. More than half the participants assumed professional roles with more leadership responsibilities since the start of the Leadership Institute.

CHLA/ABSC has developed and implemented a successful model for future Leadership Institute programs should it choose to mount similar initiative in the near future.

## Footnotes

2 Steering committee members included: Sandy Iverson (St.Michael’s Hospital, Toronto), Jennifer McKinnell (McMaster University, Hamilton), Juanita Richardson (The Michener Institute) Miriam Ticoll (Consultant) and Tim Tripp (University Health Network, Toronto).

3 Patty Fink (Northern Ontario School of Medicine) and Obianajou Mollel (Alberta Health Services)
