# Supplementary material for: Creating change through leadership development: an overview of the 2019-2021 Canadian Health Libraries Leadership Institute
Source: J Can Health Libr Assoc. 2024 Apr 1;45(1):52–6. doi: 10.29173/jchla29755 (PMC11081116; doi:10.29173/jchla29755)
Supplement: Supplementary file 1 — Supplement Appendix 1 [file JCHLA-45-052-s001.pdf]

Appendix 1: CHLA Leadership Institute Proposal

Leadership Institute for Library & Information Professionals in Canada's Health Sector

Contents

Leadership Institute for Library & Information Professionals in Canada's Health Sector 1
Introduction 1
Learning Outcomes 2
Budget 3
Overview of budget scenarios 4
Expenses Detail: Kick off event 5
Expenses detail: Capstone Event 6
Expenses Detail – Other / Fixed Costs 7
Appendix 1: Fundraising Plan 8
Industry Partnership Proposal for the CHLA / ABSC Leadership Institute 10
Appendix 2 – Promotional Material 13

Introduction

Libraries in the health sector in Canada have a vital role to play as partners in delivering the best possible health related services and supports to our citizens. We want to ensure that libraries embedded in health organizations continue to be valued as these organizations embrace and adapt to change.

To strengthen the leadership capacity of health librarians and to ensure that they are leaders in the vanguard of innovation we are mounting a leadership institute. The goals of the leadership institute are to:

- Identify a cohort of librarians with leadership potential who are committed to developing the skills that will allow them to succeed in a rapidly changing environment
- Enable individuals to identify the strengths and gaps in their knowledge/expertise and provide the tools and resources they need to help address these gaps.
- Facilitate a complex understanding and strategic approach to anticipating and meeting changing information needs in the coming five to fifteen years and to identify roles for librarians.
- Foster and encourage the development of mentor/mentee relationships between health librarians in senior positions and sustainable collegial relationships between participants.

With digitization and ubiquitous access to "information" on the internet librarians are challenged to ensure that library services are relevant and continue to add value. Libraries in certain sectors are thriving. Public libraries, where adequately funded, are flourishing as their leaders develop new and creative approaches to providing information services in public spaces. In the academic sector, libraries are becoming more deeply engaged new initiatives involving digital resource management, research data management and research support.

Libraries in hospitals and other non-profit health-related organizations face some unique challenges. Frequently under resourced, these libraries cannot provide leadership growth opportunities for their staff. Institutional mergers in the health sector are ongoing and library leaders often struggle to position themselves to be of obvious service in newly structured organizations.

A new health information landscape is on our doorstep: health care is becoming increasingly data driven and advances in artificial intelligence and personal medicine will bring many more changes. With a cohort of leaders able to navigate the evolving information and health landscapes, Canadian health librarians will be positioned to continue to make significant contributions to informed, evidence-based decision making.

The CHLA Leadership Institute is designed as a one year program that begins with a 1.5 day workshop in June of 2019 and culminates with a 1 day capstone event in 2020. In between these two events, between 8-10 webinars/meetings will be held with all participants.

Learning Outcomes

The Leadership Institute will draw on a variety of well-established frameworks for leadership, including:

- The LEADS framework promoted by the Canadian College of Health Leaders
- The Five Mind-Sets for Management developed by Henry Mintzberg, renowned management researcher and professor at McGill and Harvard Universities

These frameworks will inform the curriculum to develop leaders who will be comfortable navigating Canada's increasingly complex health information environment. The Institute's curriculum and instructors will support participants to develop their capacity to understand themselves, the organizations in which they work, and the broader context in which health care policy is developed and delivered. These skills will help the participants work more effectively to manage themselves, lead others and contribute to the mission of their employers.

Specifically, the Leadership Institute will build the capacity of participants to develop an approach to leadership that integrates the following five components:

1. *Understanding of self*: how perceptions, beliefs, tendencies affect behaviour and workplace performance, and in light of these, the most effective strategies for leveraging strengths.
2. *Context:* how to think strategically about the current and emerging environments in which organizations operate (both parent organization and departments); how to

develop action plans to continuously monitor and respond to external changes that have an impact on the internal operations of organizations.
3. *Organization:* analysis of functions required for information services in organizations in the health sector, with particular attention to the critical success factors of the workplace; how to align library services to these success factors. Participants will become equipped to articulate and use their organization's values to enhance engagement with the library.
4. *Relationships :* how to develop and implement strategies to enable multi-directional influence in organizations; this will be enhanced by experiential learning through virtual and face-to-face collaboration over the course of the Institute.
5. *Change*: participants will be guided to embrace change as constant and also appreciate that change and continuity go hand-in-hand. Participants will be encouraged to regularly monitor their environment, shape strategies responsive to evolving trends, and develop action plans for facilitating the active contribution of others in envisioning and implementing organizational and systematic strategic changes. They will understand how to better position themselves to help lead change in their organizations.

Additional key outcomes for participants include:

a. Close networks of colleagues with whom they've worked throughout the Institute who are mutually eager to build skills and experiences for leadership and senior management positions
b. Increased knowledge of current best practices in leadership and management as well as improved analysis of current industry trends affecting our field such as AI, personalized medicine, IT integration, etc.

Instruction sessions will be interactive. Participants will engage in learning through case studies, readings, guest presentations, group sharing and an individual capstone project of the participant's choosing.

Budget

This budget is designed to be self-sustaining with the majority of the costs being offset by registration fees. Should our fundraising efforts be more successful than anticipated profits can be retained by CHLA. If our fundraising efforts fall short, CHLA would ultimately be liable for that shortfall.

This budget is presented in the following parts:

1) Overview of budget scenarios depending on # of participants
2) Expense details (for the maximum size):
   a) Kick off event costs (Spring 2019)
   b) Capstone event costs (Spring 2020)
   c) Other
3) Income details (for the maximum size)

4) Appendix: Fundraising Plan

Notes & Assumptions:

- Mentors and facilitators (with the exception of one paid facilitator) will be expected to pay for their own transportation to the events but all other costs for mentors and facilitators will be absorbed.
- The venue for the Spring 2019 Kick-off event has been chosen. It is an event space 20 minutes from downtown Ottawa, and will take place June 2-4, 2019. The venue is called Manoir de la Foret. Their quote was substantially lower than any other spaces explored and is priced 'all inclusive' meaning meeting spaces, AV, food and accommodation is all included in the cost per person/room. Accommodations will be primarily shared double rooms. Our group will have exclusive use of the space.
- The venue for the 2020 Capstone event has not been chosen. The amounts budgeted are based on data gathered from various conference hotels and are informed estimates.
- The Institute will not go ahead with less than 8 participants and 12 is the maximum number of participants we will accept.
- Registration of $2000.00 will be paid by all participants. Registration could be raised to provide a higher buffer and in that case we could go as low as 8 participants.
- Fundraising goal (donations and/or grants) is set at $10,000.00. (see Appendix 1)

Overview of budget scenarios

	**12 participants<br>5 mentors<br>3 facilitators =<br>20 people**	**10 participants<br>3 mentors<br>2 facilitators =<br>14 people**	**8 participants<br>2 mentors<br>2 facilitators =<br>12 people**
**Kick off Event (Spring 2019)**			
Accommodations & Catering (all inclusive)	$6,500.00	$4,550.00	$4,225.00
Transportation	$200.00	$200.00	$200.00
**Totals**	**$6,700.00**	**$4,750.00**	**$4,425.00**
**Capstone Event (Spring 2020)**	14 rooms	10 rooms	8 rooms
Accommodations per room (1 night) @ 225.00 (incl tax)	$3,150.00	$2,250.00	$1,800.00
Catering per person @ $125	$2,500.00	$1,750.00	$1,500.00
taxes and service on catering (30%)	$750.00	$525.00	$450.00
Meeting room rental + AV	$1,000.00	$1,000.00	$1,000.00
**Totals**	**$7,400.00**	**$5,525.00**	**$4,750.00**

Other			
Program Facilitator Contract (incl HST)	$18,080.00	$18,080.00	$18,080.00
Facilitator travel costs	$400.00	$400.00	$400.00
Miscellaneous administrative fees (copying etc)	$500.00	$500.00	$500.00
**Totals**	**$18,980.00**	**$18,980.00**	**$18,980.00**
**TOTAL EXPENSES**	**$33,080.00**	**$29,255.00**	**$28,155.00**
Registration income ($2000.00 each)	$24,000.00	$20,000.00	$16,000.00
Expected Fundraising	$10,000.00	$10,000.00	$10,000.00
**BALANCE**	**$920.00**	**$745.00**	**-$2,155.00**
**RISK: (Balance without fundraising)**	**$9,080.00**	**$9,255.00**	**$12,155.00**

OPTIONS

a) **INCREASED TUITION OPTION**

**TOTAL EXPENSES**	**$28,080.00**	**$28,080.00**	**$28,080.00**
Registration income ($2250.00 each)	$24,000.00	$22,500.00	$18,000.00
Expected Fundraising	$10,000.00	$10,000.00	$10,000.00
**BALANCE**	**$5,920.00**	**$4,420.00**	**-$80.00**

OR

b) Only run with a minimum of 10 participants.

Expenses Detail: Kick off event

Notes and Assumptions:

All budget numbers in this detailed view are based on 20 people participating. Participants include mentors, facilitators, and mentees. 20 people would be approximately 12 mentees; 5 mentors; 3 facilitators.

Venue: Manoir de la Foret

Schedule of payments

Venue:

- $500.00 upon contract signing (October 2018)

- 50% ($2859.04) due 180 days prior to the event (December 4, 2018)
- Balance paid and numbers confirmed 30 days prior to the event (May 3, 2019)

Facilitator:

- 1/3 upon signing (December 2018)
- 1/3 upon completion of the June 2019 event
- 1/3 upon completion of the June 2020 event

Transportation costs: $200.00 has been budgeted to contract a taxi/van service to make group trips to pick up participants at the train station and/or airport. It is presumed that some participants may drive themselves (parking on site is free). Individual taxi fare from the airport to the venue is approximately $70.00 so we have opted to propose to arrange group transportation. Participants will be responsible for arranging their own transportation back to downtown Ottawa for the conference via shared taxis, private cars, or public transportation. Taxi fare to downtown is estimated at $40.00.

Kick off Event (Spring 2019)	
Accommodations & Catering	$6,500.00
Transportation	$200.00
**Totals**	**$6,700.00**

Expenses detail: Capstone Event

Notes and Assumptions:

All budget numbers are based on 20 people participating. Costs will be reduced should we have less than 20 people participating. Participants include both mentors and mentees. Mentors will be expected to pay for their own transportation to the events but all other costs for mentors will be absorbed. 20 people would be approximately 12 mentees; 5 mentors; 3 facilitators.

Venue: A venue for this event has not yet been selected. Therefore this budget is based on costs quoted by major hotels in Ottawa. It is assumed this event will be held in conjunction with the 2020 conference which we anticipate being held in Niagara Falls. Accommodation costs are based on major hotels quoted costs in Niagara Falls.

Note: This event is planned for one full day and one night only. Participants will be expected to make their own way to the location by 10am the morning of the event.

Capstone Event (Spring 2020)		
		**total**
**Accommodations per room** (1 night) 14 rooms Incl. tax	$225.00	**$3,150.00**
**Catering per person**	$125.00	$2,500.00
taxes and service	**30%**	$750.00
**Catering Total**		**$3,250.00**
Meeting room rental + AV		$1,000.00
**Totals**		**$7,400.00**

Expenses Detail – Other / Fixed Costs

Notes and Assumptions:

Facilitator Contract: The planning committee has selected Rebecca Jones of Dysart and Jones / PLN Focus to manage the development and delivery of the Leadership Institute. Rebecca focuses on planning, problem-solving, organizational design, and leadership coaching. Rebecca has led and facilitated over 100 planning and organizational design projects with clients from small, volunteer-run non-profits to large research university libraries. Rebecca also teaches professional development courses for the Southern Ontario Library Services' Advancing Public Library Leadership (APLL), is a program lead of BC InterLINK's Library Leadership Excellence and Development Institute (LLEAD) and was a Mentor for the Northern Exposure to Leadership Institute.

Rebecca will be supported by a Steering Committee of CHLA volunteer members as well as those selected to play mentoring roles.

Facilitator Travel: Rebecca is located in Toronto, therefore her travel costs to both Ottawa and Niagara Falls should be covered by the allotted $400.00.

Admin Fees: $500.00 has been allotted to cover any additional and unforeseen administrative costs such as copying of articles for participants.

Other / Fixed Costs	
Program Facilitator Contract (incl HST)	$18,080.00
Facilitator travel costs	$400.00
Miscellaneous administrative fees (copying etc)	$500.00
**Totals**	**$18,980.00**

Appendix 1: Fundraising Plan

In consultation with the Development Officer, the Planning Committee has developed a two-pronged fundraising plan:

1) Corporate Sponsorship (see attached partnership proposal). This proposal has been provided to 3 corporate partners so far and has received positive responses with some verbal commitments of support up to $10,000. It should be noted that we have received one verbal offer for $10,000 and two smaller verbal offers of unspecified amounts likely in the range of $500-$1000 each.

2) SSHRC Connections Grant Application. We have applied to be an accepted institution to submit a grant application to SSHRC. We have had conversations regarding our project with SSHRC staff who have assured us that our project fits with the criteria of the grant. The deadline for the grant application is November 1 and we are on track to submit. We will hear their decision in early January. This is a matching funds granting program, and we are asking for $18,840.00. The grant would facilitate paid presenters at the initial workshop; travel supplements for participants and the engagement of a student on a part time basis to assist with the organization of the initial workshop. The grant would also pay for half of the Facilitator's contract. If the grant is awarded for the full amount it would reduce our fundraising needs to $5000.

Budget with SSHRC Funding:

**EXPENSES**	
**Kick off Event (Spring 2019)**	
Accommodations & Catering (all inclusive)	$6,500.00
Participant transportation	$3,730.00
Student Staff salary	$8,320.00
Student staff transportation	$400.00
Presenter transportation	$1,800.00
**Total**	**$20,750.00**
**Capstone Event (Spring 2020)**	14 rooms
Accommodations per room (1 night) @ 225.00 (incl tax)	$3,150.00
Catering per person @ $125	$2,500.00
taxes and service on catering (30%)	$750.00
Meeting room rental + AV	$1,000.00
**Total**	**$7,400.00**
**Other**	
Program Facilitator Contract (incl HST)	$18,080.00
Facilitator travel costs	$400.00
Miscellaneous administrative fees (copying etc)	$500.00
**Total**	**$18,980.00**
**TOTAL EXPENSES**	**$47,130.00**

INCOME	
Registration income ($2000.00 each)	$24,000.00
SSHRC grant	$18,840.00
Expected Fundraising	$5,000.00
**Total**	**$47,840.00**
**BALANCE**	**$710.00**
**RISK: (Balance without fundraising)**	**-$4,290.00**

Industry Partnership Proposal for the CHLA / ABSC Leadership Institute

Who's bringing the knowledge to the table in healthcare?

The Canadian health care sector is a rapidly-changing environment – and is increasingly data driven. The challenge for senior management in health services organizations is **not the fire hose of information** enabled by technology. The challenge is access to curated knowledge to **inform evidence-based decisions** – enabled by librarians as **strategic information leaders** in the sector.

The CHLA/ABSC Leadership Institute will develop these strategic leaders to take on this challenge - and to be responsible for mobilizing knowledge and informing decisions at the most senior levels within their organizations.

The Leadership Institute is working in collaboration with Dysart & Jones to develop a customized health care sector framed curriculum and a tailored evaluation process. The Leadership Institute will be 12 months in duration – with an initial in-person 1 ½ day intensive scheduled for June 2019 (to immediately precede the CHLA/ ABSC conference in Ottawa), followed by bi-monthly coaching and touch-bases with faculty and mentors, concluding with a 1 day intensive with a capstone presentation by each participant at the subsequent CHLA/ABSC conference in 2020. We propose that our first cohort will be comprised of 8 – 10 professionals who will be selected by application.

BACKGROUND:

Why we need a CHLA/ABSC Leadership Institute: Our members asked – and we have answered

Health care in Canada is subject to an ever-changing environment and is increasingly data driven. Advances in areas such as artificial intelligence and personalized medicine will have a significant impact on how medicine is practiced in the coming years. In this rapidly-evolving environment, the energetic and creative engagement of health science librarians is imperative. These strategic information leaders must actively identify and pursue new opportunities that the changes in the health sector will present for librarians – and for all users of information.

Through the membership survey conducted in the Spring and the focus group held at annual conference in June, our members have spoken – they want – and need – to develop strategic information leadership capacity in the context of the health care sector:

New library leaders cannot rely upon the tried and true. The strategic information leaders of the health care organizations of the future must be nimble learners who are in the vanguard of innovation – professionals who are comfortable with and capable of providing information leadership.

Our goals:

☐Identify a cohort of health science librarians with strategic leadership potential who are committed to developing personal leadership skills that will allow them to succeed in a changing health information environment.

☐Enable individuals to identify the strengths and gaps in their knowledge/expertise and provide the tools and resources they need to help address these gaps.

☐Facilitate (through readings, guest speakers, lectures, group discussions, etc.) a complex understanding and strategic approach to anticipating and meeting changing health information needs in the coming five to fifteen years.

☐Foster and encourage the development of mentor/mentee relationships between health science librarians in senior leadership positions, as well as sustainable collegial and supportive relationships between participants.

☐Develop strong leadership to ensure the sustainability of our professional associations.

THE LEADERSHIP INSTITUTE:

The CHLA / ABSC Leadership Institute is designed as a one-year program that begins with a 1 ½ day workshop (Sunday June 2 – Tuesday June 4, 2019) at a venue 20 minutes outside of Ottawa, Manoir de la Forêt. The culminating 1 day (tentative: Monday June 8, 2020) capstone event will be held in Niagara Falls. In between these two events, 8 – 10 webinars / meetings will be held with the participants, faculty and mentors.

Total Expenses	Total income
2019 Workshop $6,500	Registration (proposed $2000 X 10) $20,000
2020 Capstone Event $7,175	Sponsorships TBD
Other costs $18,980	
TOTAL $32,855	GRAND TOTAL ($12,855)

THE OPPORTUNITY:

Platinum Sponsor (EXCLUSIVE): $30,000

- ✓ CO-BRANDING of the Leadership Institute with CHLA / ABSC
- ✓ Propose member of faculty
- ✓ Inform the development of curriculum

Gold Sponsor: $10,000

- ✓ Propose member of faculty
- ✓ Inform the development of curriculum

Silver Sponsor: $5,000

- ✓ Inform the development of curriculum

NOTE: This opportunity must be considered in addition to any commitment to either the 2019 or 2020 CHLA / ABSC conference.

September 2018

Appendix 2 – Promotional Material

CHLA/ABSC Leadership Institute: Now accepting applications

The Canadian Health Libraries Association/Association des bibliothèques de la santé du Canada (CHLA/ABSC) is pleased to announce the launch of its first ever Leadership Institute.

The Leadership Institute is designed for health science librarians who want to develop strategic approaches to mobilizing knowledge and enabling informed decision- making at senior levels within their organizations. The Institute will be of interest to librarians who would like to position themselves to take on leadership roles as health information professionals.

Increasingly, data is driving decision-making in health– and data is everywhere. One of the biggest challenges that healthcare practitioners and senior managers face is how to ensure that data is curated and that decisions are evidence and knowledge based. In this environment health librarians are uniquely poised to play leadership roles.

To assume positions of increasing responsibility and influence, librarians must think and act strategically. They need to:

- understand, leverage and communicate the value they bring as information professionals;
- assess and anticipate the changing knowledge/information needs of the organizations in which they work, and
- understand the broader systemic issues that have implications for both the health and library sectors.

The Steering Committee of the Leadership Institute is working in collaboration with Rebecca Jones from Dysart & Jones to develop a multi-faceted curriculum that will address these issues and support participants to develop their leadership skills.

The Institute will be 12 months in duration. It will begin with an initial in-person 1 ½ day intensive on June 2-4, 2019 (just preceding the CHLA/ ABSC conference in Ottawa), followed by bi-monthly coaching sessions with mentors and online seminars, and concluding with a 1 day intensive session with a capstone presentation at the CHLA/ABSC conference in 2020.

The initial in-person intensive will be held at the Manoir de la Forêt special events venue in Cantley, Quebec, 20 minutes from downtown Ottawa. Manoir de la Forêt is set in a 29 acre forest and offers peaceful surroundings for a unique learning experience.

Who should apply?

- Applicants must be graduates from an accredited library and information studies program.

- Applicants must currently be employed in a health information environment.

- Applicants must have a minimum 5 years of post-graduate working experience, of which at least 2 years has been in a health environment.
- Ideally Applicants should have had some experience leading people or projects in either a work or volunteer capacity.
- The Applicant's record should indicate successful employment experience, and leadership potential.
- Applicants must be able to participate in all sessions of the Institute.
- Applicants must be a member in good standing of CHLA/ABSC.
- Applicants will be responsible for ensuring they have the technological capacity to fully engage in the virtual meetings with a device that has full camera and audio capabilities.
- Applicants should be prepared for a significant time commitment (1.5 days in June 2019; approximately 4 hours per month and 1 day in May/June 2020), and have the support of their library and/or supervisor to participate.

How is the program structured?

The program is structured in 3 parts, with 4 components:

1. **The initial intensive**. This 1.5 day retreat will be held June 2-4, 2019 and is designed to introduce participants to the core curriculum, build solid group dynamics with other participants, instructors and mentors, and explore current and emerging challenges facing information leaders in the Canadian healthcare sector.

2. **Ongoing throughout the year:**

    a. **Tele-meetings.** Approximately 8 online meetings (60-90 minutes each) will be scheduled over the course of the year. These online meetings may include short didactic presentations by leaders in health or health information services to assist participants to apply the strategic leadership skills they have learned to specific health workplace examples, followed by group discussions or homework exercises.

    b. **Ongoing mentoring.** All participants will be matched with a mentor. Mentors will meet either online or in-person with participants 6-8 times over the course of the year. Participants may choose to discuss any or all of the following with their mentors:
        - Current career challenges
        - Developing their capstone project
        - Materials covered during the tele-meetings
        - Their understanding of any of the issues or readings raised during the online meetings or during the initial intensive

- Any other professional questions or concerns

3. **Capstone event.** This 1 day retreat will primarily consist of presentations of capstone projects with structured, constructive feedback.

Program Curriculum Details

The Leadership Institute will draw on a variety of well-established frameworks for leadership, including:

- The LEADS framework promoted by the Canadian College of Health Leaders
- The Five Mind-Sets for Management developed by Henry Mintzberg, renowned management researcher and professor at McGill and Harvard Universities

These frameworks will inform the curriculum to develop leaders who will be comfortable navigating Canada's increasingly complex health information environment. The Institute's curriculum and instructors will support participants to develop their capacity to understand themselves, the organizations in which they work, and the broader context in which health care policy is developed and delivered. These skills will help the participants work more effectively to manage themselves, lead others and contribute to the mission of their employers.

Specifically, the Leadership Institute will build the capacity of participants to develop an approach to leadership that integrates the following five components:

6. *Understanding of self*: how perceptions, beliefs, tendencies affect behaviour and workplace performance, and in light of these, the most effective strategies for leveraging strengths.
7. *Context:* how to think strategically about the current and emerging environments in which organizations operate (both parent organization and departments); how to develop action plans to continuously monitor and respond to external changes that have an impact on the internal operations of organizations.
8. *Organization:* analysis of functions required for information services in organizations in the health sector, with particular attention to the critical success factors of the workplace; how to align library services to these success factors. Participants will become equipped to articulate and use their organization's values to enhance engagement with the library.
9. *Relationships :* how to develop and implement strategies to enable multi-directional influence in organizations; this will be enhanced by experiential learning through virtual and face-to-face collaboration over the course of the Institute.
10. *Change*: participants will be guided to embrace change as constant and also appreciate that change and continuity go hand-in-hand. Participants will be encouraged to regularly monitor their environment, shape strategies responsive to evolving trends, and develop action plans for facilitating the active contribution of others in envisioning and implementing organizational and systematic strategic changes. They will understand how to better position themselves to help lead change in their organizations.

Participants in the Leadership Institute will develop a close network of colleagues with whom they've worked throughout the Institute, all of whom who are eager to build skills and experiences for leadership and senior management positions.

Instruction sessions will be interactive. Participants will engage in learning through case studies, readings, guest presentations, group sharing and an individual capstone project of the participant's choosing.

Each project will address an organizational need involving stakeholders and/or partners in myriad departments or functions. It will provide an opportunity for the participant to purposefully use the five components– individually and holistically. Capstone projects should offer practical solutions to real situations, and while the project might be research based, it should result in something that can be implemented and used in the workplace. Participants are encouraged to design their projects in collaboration with their workplace leaders. These are two examples of capstone projects:

- Identify the key performance indicators (KPI's) of the hospital and in consultation with supervisor and hospital administration staff, determine which of these are of greatest relevance to the library. Work with library staff to structure statistics and operating metrics that conform with the KPI's used by the hospital. Consider how this process might also be applied to other hospital departments. Present measurement system in write-up to hospital admin staff with a view to how systems thinking of this type can be more generalized throughout the hospital, i.e. how statistics can be gathered through the lenses of the hospital's operating metrics and strategic KPI's.

- Design a data (or other) service for your library, taking into consideration local constraints like funding, staffing. Work with supervisor and other key library staff as appropriate. Present strategies for overcoming challenges.

How to apply:

Please send an application package including the following to: leadinst@chla-absc.ca before midnight November 11, 2018.

1. Cover letter (no more than **two pages**) should address these specific questions:
    - Why are you interested in attending the Institute?
    - What are some of the challenges you are facing as an emerging leader and how will the Institute benefit your work?
    - How do you expect that your particular skills, experience and perspective will contribute to the Leadership Institute?

    AND state:
    - Your willingness to assume transportation costs, the registration fee and any incidental accommodation expenses.

2. A letter of support from your employer, a library-related organization or association or peer in the library sector attesting to your readiness and ability to be a leader in health information services

3. A résumé or curriculum vitae. (max 3 pages)

How does the Selection Process work?

Acceptance to the CHLA/ABSC Leadership Institute is made through a competitive application process. The selection committee, composed of **3-5** senior members of CHLA/ABSC, seeks to create a participant group with diverse experience, background, and perspectives.

The goal of the CHLA/ABSC Leadership Institute is to prepare participants for a depth and breadth of experiences that can be leveraged to advocate for, initiate and lead change as the health information landscape evolves. Successful applications to the Institute will include polished, professional cover letters, and strong letters of support. Through the application, the selection committee seeks to learn an applicant's background and how they see themselves contributing as leaders in the health information community in the future.

In addition to selecting applicants with leadership interests and potential, the selection committee will strive to ensure that the final selected cohort will reflect geographic, cultural and gender diversity.

A minimum of 8 participants are required for the Leadership Institute to be viable. The maximum number of participants that will be accepted is 12.

How much does the program cost?

There are two major costs associated with participation in the Leadership Institute: tuition and transportation.

Tuition is paid to CHLA/ABSC and supports the cost of participation, food and accommodation (shared double occupancy) at both the initial 1.5 day (2 night) intensive and the final 1 day (1 night) capstone event. The fee also covers all online events and mentoring sessions.

Participants will be required to bring their results of a personality assessment exercise (Myers Briggs, DISC, or other). Participants who have not already completed one of these within the last 5 years will need to complete one and some costs may be involved (approximately $75.00)

Participants are responsible for transportation expenses to and from the site of the institute (Ottawa, 2019 and Niagara Falls, 2020)

Participants should plan to attend the annual CHLA/ABSC conferences in 2019 and 2020

2019/20 Tuition Rates: $2000.00 CDN. 50% is due within 2 weeks of receiving confirmation of acceptance in the Institute and is non-refundable. The remaining 50% is due on May 5th, 2019 and is non-refundable.

Participants will be eligible to apply for up to $500.00 from CHLA/ABSC Professional Development Grants and/or CHLA/ABSC Rural and Remote Opportunities Grants.

Note about language:

Unfortunately, the Leadership Institute will be conducted solely in English. Any Francophone applicants should be aware of this. However, should any participants prefer a French speaking mentor all attempts will be made to recruit a mentor capable of conversing professionally and fluently in French. Please note this request in your application package.

Seeking support from your institution?

We have drafted the following sample letter to help you think about how you might approach your management for support to participate in the Leadership Institute. If you have any other questions or concerns please write to leadinst@chla-absc.ca and a member of the LI Steering Committee will contact you.

Dear *Manager,*

The Canadian Health Libraries Association / Association des bibliothèques de la santé du Canada (CHLA/ABSC) has recognized the urgent need to provide leadership training opportunities to information professionals working in the healthcare sector and is launching its first ever Leadership Institute beginning in June of 2019.

The Institute is designed to take place over the course of a year and includes at least 35 hours of structured learning. The first 1.5 days will offer an intensive residential learning experience next June (June 2-4, 2019) in Ottawa. Over the course of the subsequent twelve months, participants will engage in regular tele-meetings and 1-1 mentoring sessions. The program will culminate with a 1 day in-person meeting where all participants will present their capstone projects.

A key part of the curriculum is that each participant must engage in a 'capstone' project. These projects should provide an opportunity for participants to investigate or practice leadership while also addressing an organizational need, involve stakeholders and/or partners and offer practical solutions to real situations. Some ideas I have for a capstone project that I believe would benefit our institution are: *insert ideas here*.

At $2000.00, including meals and accommodations at both the kick-off and capstone in-person meetings, the tuition fee for the Leadership Institute is extremely competitive and far less than most library leadership programs. For example, the SPARC Open Education Leadership Program is a minimum of $3200.00 and the Harvard Leadership Institute for Academic Librarians is $3800.00. The CHLA/ABSC Leadership Institute is the only Canadian based leadership program for library and information professionals in the health sciences and offers exceptional value.
