# Supplementary material for: Creating change through leadership development: an overview of the 2019-2021 Canadian Health Libraries Leadership Institute
Source: J Can Health Libr Assoc. 2024 Apr 1;45(1):52–6. doi: 10.29173/jchla29755 (PMC11081116; doi:10.29173/jchla29755)
Supplement: Supplementary file 2 — Supplement Appendix 2 [file JCHLA-45-052-s002.pdf]

Appendix 2: CHLA Leadership Institute Call for Applications

CHLA/ABSC LEADERSHIP INSTITUTE

Now Accepting Applications!

The Canadian Health Libraries Association /Association des bibliothèques de la santé du Canada (CHLA/ABSC) is pleased to announce the launch of its first ever Leadership Institute.

The Leadership Institute is designed for health science librarians who want to develop strategic approaches to mobilize knowledge and enable informed decision-making at senior levels within their organizations. The Institute will be of interest to librarians wishing to position themselves for senior leadership roles in the health sector.

Increasingly, data is driving decision-making in health; and data is everywhere. One of the biggest challenges facing healthcare practitioners and senior managers is how to ensure that data is curated, and that decisions are based on evidence and explicit knowledge. In this environment, health librarians are uniquely poised for leadership roles.

To assume positions of increasing responsibility and influence, librarians must think and act strategically. They must:

- understand, leverage and communicate the value they bring as information professionals;
- assess and anticipate the changing knowledge/information needs of the organizations in which they work; and
- understand the broader systemic issues that have implications for both the health and library sectors.

The Steering Committee of the Leadership Institute is working in collaboration with Rebecca Jones from Dysart & Jones to develop a multi-faceted curriculum that will address these issues and support participants to develop their leadership skills.

The Institute is twelve months in duration. Beginning with an initial in-person 1½ day intensive on June 2-4, 2019 (preceding the CHLA/ABSC conference in Ottawa), participants progress through bi-monthly coaching sessions with mentors and online seminars, and the Institute concludes with a full day session with capstone presentations at the CHLA/ABSC conference in 2020.

The initial in-person intensive will be held at the Manoir de la Forêt special events venue in Cantley, Quebec, 20 minutes from downtown Ottawa. Manoir de la Forêt is set in a 29 acre forest and offers peaceful surroundings for a unique learning experience.

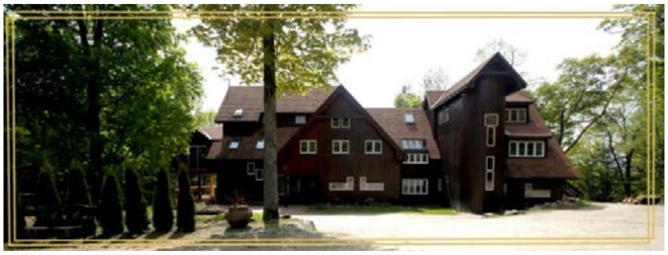

Manoir de la Forêt

How is the program structured?

The program is structured in 3 parts, with 4 components:

1. **The initial intensive**. This 1.5 day retreat will be held June 2-4, 2019 and is designed to introduce participants to the core curriculum, build solid group dynamics with other participants, instructors and mentors, and explore current and emerging challenges facing information leaders in the Canadian health sector.
2. **Ongoing throughout the year:**
   a. **Tele-meetings.** Approximately 8 online meetings (60-90 minutes each) will be scheduled over the course of the year. These online meetings will include presentations by leaders in health or information services to assist participants to apply the leadership skills they have learned to specific workplace scenarios, and will be followed by group discussions and/or homework exercises.

   b. **Mentoring.** All participants will be matched with a mentor. Mentors will meet either online or in-person with participants 6-8 times over the course of the year. Participants may choose to discuss any or all of the following with their mentors:
      - Current career challenges
      - Developing their capstone project
      - Materials covered during the tele-meetings
      - Their understanding of any of the issues or readings raised during the tele-meetings or the initial intensive
      - Any other professional questions or concerns
3. **Capstone event.** This full day retreat will primarily consist of presentations of capstone projects with structured, constructive feedback.

Who should apply?

- Applicants must be graduates from an accredited library and information studies program.
- Applicants should have a minimum of 4 years post-graduate working experience, of which at least 2 years must be in a health environment. Ideally, applicants will have had some experience leading people or projects in either a work or volunteer capacity.
- The applicant's record should indicate successful employment experience and leadership potential.
- Applicants will be responsible for ensuring they have the technological capacity to fully engage in the virtual meetings with a device that has full camera and audio capabilities.
- Applicants must be a member in good standing of CHLA/ABSC.
- Applicants are expected to attend all sessions of the Institute and should be prepared for a significant time commitment (1.5 days in June 2019; approximately 4 hours per month and 1 day in May/June 2020), and have the support of their library and/or supervisor to participate.

Program curriculum details

The Leadership Institute will draw on a variety of well-established frameworks for leadership, including:

- The LEADS framework promoted by the Canadian College of Health Leaders
- The Five Mind-Sets for Management developed by Henry Mintzberg, renowned management researcher and professor at McGill and Harvard Universities

These frameworks inform the curriculum to develop leaders who will be comfortable navigating Canada's increasingly complex health information environment. The Institute's curriculum and instructors will support participants to develop their capacity to understand themselves, the organizations in which they work, and the broader context in which health care policy is developed and delivered. These skills will help the participants work more effectively to manage themselves, lead others and contribute to the mission of their employers.

Specifically, the Leadership Institute will build the capacity of participants to develop an approach to leadership that integrates the following five components:

1. ***Understanding of self***: how perceptions, beliefs and tendencies affect behaviour and workplace performance, and effective strategies for leveraging strengths in light of these factors.
2. ***Context:*** how to think strategically about the current and emerging environments in which organizations operate (both parent organization and departments); how to develop action plans to continuously monitor and respond to external changes that have impact on the internal operations of organizations.
3. ***Organization:***
analysis of functions required for information services in organizations in the health sector, with particular attention to the critical success factors of the workplace and how to align library services to these success factors. Participants will become equipped to articulate and use their organization's values to enhance engagement with the library.
4. ***Relationships:*** how to develop and implement strategies to enable multi-directional influence in organizations; this will be enhanced by experiential learning through virtual and face-to-face collaboration over the course of the Institute.
5. ***Change***: participants will be guided to embrace change as constant and also appreciate that change and continuity go hand-in-hand. Participants will be encouraged to regularly monitor their environment, shape strategies responsive to evolving trends, and develop action plans to facilitate the active contribution of others in envisioning and implementing organizational and systematic strategic changes. They will understand how to better position themselves to help lead change in their organizations.

Participants in the Leadership Institute will develop a close network of colleagues with whom they will have collaborated throughout the Institute, all of whom are eager to build skills and experiences for leadership and senior management positions.

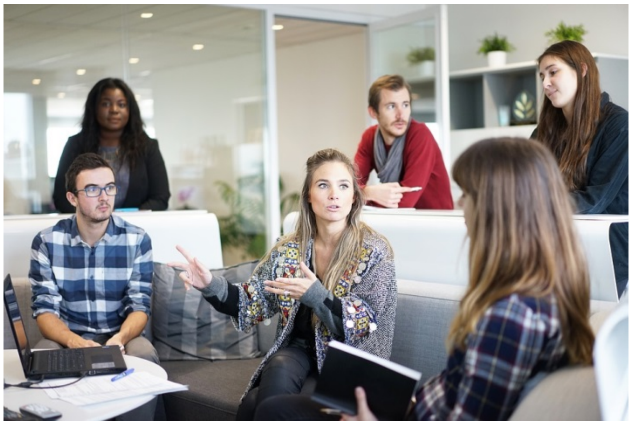

Instruction sessions will be interactive and participants will engage in learning through case studies, readings, guest presentations, group sharing and an individual capstone project of the participant's choosing.

Each capstone project will address an organizational need involving stakeholders and/or partners in a variety of departments or functions. It will provide an opportunity for the participant to purposefully use the five components– individually and holistically. Capstone projects should offer practical solutions to real situations. While projects might be research based, they should result in something that can be implemented and used in the workplace. Participants are encouraged to design their projects in collaboration with their workplace leaders. Here are two examples of capstone projects:

A. *Identify the key performance indicators (KPIs) of the hospital and in consultation with supervisor and hospital administration staff, determine which of these are of greatest relevance to the library. Work with library staff to structure statistics and operating metrics that conform with the KPIs used by the hospital. Consider how this process might also be applied to other hospital departments. Present the measurement system to hospital admin staff with a view to how systems thinking of this type can be more generalized throughout the hospital, i.e. how statistics can be gathered through the lenses of the hospital's operating metrics and strategic KPIs.*

B. *Design a data (or other) service for your library, taking into consideration local constraints like funding and staffing. Work with supervisor and other key library staff as appropriate. Present strategies for overcoming challenges.*

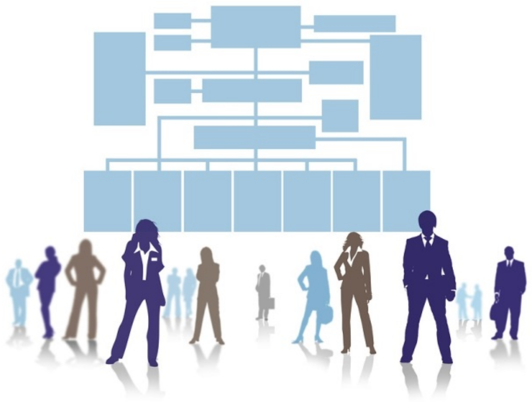

How to apply:

Please send an application package including the following to: leadinst@chla-absc.ca before midnight **November 11, 2018.**

1. Cover letter (no more than **2 pages**) should address these specific questions:
   - Why are you interested in attending the Institute?
   - What are some of the challenges you are facing as an emerging leader and how will the Institute benefit your work?
   - How do you expect that your particular skills, experience and perspective will contribute to the Leadership Institute?

   AND state:

   - Your willingness to assume transportation costs, the registration fee and any incidental accommodation expenses.
2. A letter of support from your employer, a library-related organization or association or peer in the library sector attesting to your readiness and ability to be a leader in health information services.
3. A résumé or curriculum vitae. (max **3 pages**)

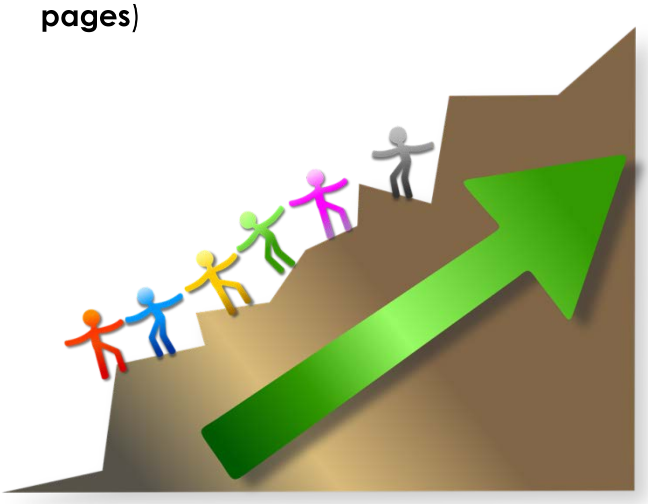

How does the selection process work?

Acceptance to the CHLA/ABSC Leadership Institute is made through a competitive application process. The selection committee, composed of 3-5 senior members of CHLA/ABSC, seeks to create a participant group with diverse experience, background, and perspectives.

Successful applications to the Institute will include polished, professional cover letters, and strong letters of support. Through the application, the selection committee seeks to learn an applicant's background and how they see themselves contributing as leaders in the health information community in the future.

In addition to selecting applicants with leadership interests and potential, the selection committee will strive to ensure that the final selected cohort will reflect geographic, cultural and gender diversity.

Only10-12 applicants will be selected.

How much does the program cost?

Registration fees (paid to CHLA/ABSC) cover the full year of sessions including food and accommodation (shared double occupancy) at both the initial 1.5 day (2 night) intensive and the final full day (1 night) capstone event.

Participants will be required to bring their results of a personality diagnostic (Myers Briggs, DISC, or other). Participants who have not already completed one of these within the last 5 years will need to complete one and some costs may be involved (approximately $100.00).

Participants are responsible for transportation expenses to and from the site of the Institute (Ottawa, 2019 and Niagara Falls, 2020)

Participants should plan to attend the annual CHLA/ABSC conferences in 2019 and 2020.

2019/20 Rates:

$2000.00 CDN. 50% is due within 2 weeks of receiving confirmation of acceptance to the Institute and is non-refundable. The remaining 50% is due on May 4th, 2019 and is non-refundable.

Participants will be eligible to apply for CHLA/ABSC Professional Development Grants and/or CHLA/ABSC Rural and Remote Opportunities Grants.

Note about language:

The Leadership Institute will be conducted solely in English. However, should any participants prefer a French speaking mentor, all attempts will be made to recruit a mentor capable of conversing professionally and fluently in French. Please note this request in your application package.

Seeking support from your institution?

We have drafted the following sample letter to help you think about how you might approach your management for support to participate in the Leadership Institute.

If you have any other questions or concerns please write to leadinst@chla-absc.ca and a member of the Leadership Institute Steering Committee will contact you.

Dear *Manager,*

The Canadian Health Libraries Association / Association des bibliothèques de la santé du Canada (CHLA/ABSC) has recognized the urgent need to provide leadership training opportunities to information professionals working in the healthcare sector and is launching its first ever Leadership Institute beginning in June of 2019.

The Institute is designed to take place over the course of a year and includes at least 35 hours of structured learning. The first 1.5 days will offer an intensive residential learning experience next June (June 2-4, 2019) in Ottawa. Over the course of the subsequent twelve months, participants will engage in regular tele-meetings and 1-1 mentoring sessions. The program will culminate with a 1 day in-person meeting where all participants will present their capstone projects.

A key part of the curriculum is that each participant must engage in a 'capstone' project. These projects should provide an opportunity for participants to investigate or practice leadership while also addressing an organizational need, involve stakeholders and/or partners and offer practical solutions to real situations. Some ideas I have for a capstone project that I believe would benefit our institution are: *insert ideas here*.

At $2000.00, including meals and accommodations at both the kick-off and capstone in-person meetings, the tuition fee for the Leadership Institute is extremely competitive and far less than most library leadership programs. For example, the SPARC Open Education Leadership Program is a minimum of $3200.00 and the Harvard Leadership Institute for Academic Librarians is $3800.00. The CHLA/ABSC Leadership Institute is the only Canadian based leadership program for library and information professionals in the health sciences and offers exceptional value.
