# Supplementary material for: Creating change through leadership development: an overview of the 2019-2021 Canadian Health Libraries Leadership Institute
Source: J Can Health Libr Assoc. 2024 Apr 1;45(1):52–6. doi: 10.29173/jchla29755 (PMC11081116; doi:10.29173/jchla29755)
Supplement: Supplementary file 3 — Supplement Appendix 3 [file JCHLA-45-052-s003.pdf]

Appendix 3: CHLA Leadership Institute Curriculum

CHLA/ABSC LEADERSHIP INSTITUTE 2019-2020

The Curriculum

Curriculum development took place over the winter and spring of 2019. It was an iterative process, led by Rebecca Jones with a lot of input from the Steering Committee.The Leadership Institute drew on a variety of well-established frameworks for leadership, including most notably:

- The LEADS framework promoted by the Canadian College of Health Leaders
- The Five Mind Sets for Management developed by Henry Mintzberg, renowned management researcher and professor at McGill and Harvard Universities

The text below, taken from the LI promotional brochure, describes the approach/framework:

The Institute's curriculum and instructors will support participants to develop their capacity to understand themselves, the organizations in which they work, and the broader context in which health care policy is developed and delivered.

Specifically, the Leadership Institute will build the capacity of participants to develop an approach to leadership that integrates the following five components:

1. Understanding of self

How perceptions, beliefs and tendencies affect behaviour and workplace performance, and effective strategies for leveraging strengths in light of these factors.

2. Context

How to think strategically about the current and emerging environments in which organizations operate (both parent organization and departments); how to develop action plans to continuously monitor and respond to external changes that have impact on the internal operations of organizations.

3. Organization

Analysis of functions required for information services in organizations in the health sector, with particular attention to the critical success factors of the workplace and how to align library services to these success factors. Participants will become equipped to articulate and use their organization's values to enhance
engagement with the library.

4. Relationships

How to develop and implement strategies to enable multi-directional influence in organizations; this will be enhanced by experiential learning through virtual and face-to-face collaboration over the course of the Institute.

5. Change

Participants will be guided to embrace change as constant and also appreciate that change and continuity go hand-in-hand. Participants will be encouraged to regularly monitor their environment, shape strategies responsive to evolving trends, and develop action plans to facilitate the active contribution of others in envisioning and implementing organizational and systematic strategic changes. They will understand how to better position themselves to help lead change in their organizations.

The format for the Leadership Institute involved two residential sessions (one at the beginning and one at the end of the Institute), plus 8-10 virtual sessions. The participants were expected to: attend and actively participate in the sessions, read assigned articles, work on their individual capstone projects and report on their progress to the group. They were required to deliver a final presentation on their project to the group and mentors.

Virtual Sessions

Following the initial residential session, eight virtual learning sessions took place. Information gathered during the focus groups held the prior year informed the topics selected. Members of the Steering Committee, mentors, and the program facilitator helped identify and recruit speakers, thus distributing the workload across the entire planning group. While eight virtual events were scheduled (one per month with time off over the summer and December), only six topics were selected. This approach was intentional, leaving gaps for Institute participants to propose new ideas as their learning evolved. Sessions 6 (Inclusive Workplaces) and Session 8 (Open Q&A) were the two additional topics added later in the planning year.

The following online Sessions ran monthly between August 2019 and May 2020

Session 1: Health libraries: funding, engaging health care providers & engaging patients.
• Miriam Ticoll, BA, MLS, moderator.
• Tim Tripp, BSc, MLIS (Director of Library and Information Services at the University Health Network)– The Clinical Perspective – Engaging Healthcare Providers.
• Jennifer McKinnell, BA, MA, MLIS (Director at McMaster University’s Health Sciences Library – The Academic Perspective – the Funding Realities
• Laura Williams, MSW, RSW, CHE (Director, Patient Engagement at the University Health Network) – The Non-Library Patient Experience

Session 2: Artificial Intelligence in the Health Sector: Implications for libraries and information management
• Mike Ridley, Librarian Emeritus, Guelph; PhD Candidate, Western; Visiting Scholar; Ryerson; Postgraduate Affiliate, Vector Institute

Session 3: Health Professions Education in Canada
• Dr. Brian Hodges, Exec VP Education and Chief Medical Officers, UHN

Session 4: Developing Visions & Actions: Engaging All
• Mandy Lowe, Senior Director, Clinical Education, University Health Network

Session 5: Inclusive Workplaces
• Shannon Jones, Director of Libraries, Medical University of South Carolina.
• Dr. Shailoo Bedi, Director, Office of Student Academic Success, Division of Learning, Teaching Support & Innovation (LTSI); Adjunct Assistant Professor, Educational Psychology & Leadership Studies; University of Victoria, and Director, Academic Commons & Strategic Assessment, University of Victoria Libraries.

Session 6: Leaders & Research: what to ask and how to find answers
• Joan Bartlett, Associate Professor @ McGill University, School of Information Studies

Session 7: Research Data Management (RDM): implications for you and our clients in the hospital, clinical and academic settings?
• Jean Shipman, Vice President, Global Library Relations (retired), Elsevier
• Rebecca Morin, Head of Research & Instruction, Hirsh Health Sciences Library, Tufts University

Session 8: Speaker: Burning Questions
• Mentors and Instructors: This was an open session in which mentors and instructors addressed the cohorts' "burning questions.

Closing Session

The original intention was for the Leadership Institute to conclude with a one-and-a-half-day residential session to be held in Niagara Falls immediately preceding the 2020 CHLA/ABSC Conference. The plan for the closing session was that each participant would present their capstone project and that there would be time for evaluation, reflection, and a 'graduation' ceremony.

Unfortunately, due to the global COVID19 pandemic, the CHLA/ABSC 2020 conference was cancelled. Many Institute participants found themselves unable to complete their capstone projects on schedule due to pandemic related stresses as their institutions struggled to address the new realities. The Steering Committee consulted the participants on how they wanted to proceed and with this input they proposed the following:
• Participants who were ready to present their capstone projects would do so during online sessions scheduled in September or November (no one opted for November and this session was cancelled)
• The closing residential session would be planned in conjunction with the 2021 conference in Winnipeg and would include remaining capstone presentations, graduation, evaluation, reflection and a guest speaker

The CHLA/ABSC Board decided that the 2021 CHLA/ABSC would be delivered online due to COVID precautions. At that point the Steering Committee, in consultation with the participants, set a date for an online closing session for the Leadership Institute. This session took place on April 23, 2021. The agenda for that session included:
• Remaining capstone presentations
• Participant round table: Each participant provided brief updates on their capstone projects and professional and personal lives
• Reflections from Mentors, Facilitator, and Steering Committee Members
• Graduation Ceremony
