# Supplementary material for: Creating change through leadership development: an overview of the 2019-2021 Canadian Health Libraries Leadership Institute
Source: J Can Health Libr Assoc. 2024 Apr 1;45(1):52–6. doi: 10.29173/jchla29755 (PMC11081116; doi:10.29173/jchla29755)
Supplement: Supplementary file 4 — Supplement Appendix 4 [file JCHLA-45-052-s004.pdf]

Appendix 4: Learning Plan

Learning Plan:

Starting Point

When we decide to learn and develop new skills and knowledge, we need to plan our approach and take an honest look at our starting point. It doesn't matter if we are learning about a sport, using a 3D printer to make phone case or developing our capabilities as a manager, we need a plan that beings "here" and has a goal of "there".

This tool is designed to help you determine your starting point regarding the Institute's goals and each of five "mindsets" (Mintzberg) or "domains" (LEADS). Knowing your starting point is essential for you to chart a Learning Plan that you'll use throughout the Institute. This tool is not designed to convey where you are "weak" or "strong" but rather to identify those capabilities and areas in which you demonstrate knowledge and confidence, and those areas to which you may want to give more attention or determine strategies for better managing.

This tool, the mindsets and domains upon which it is based, is thought-provoking. It is designed to initiate consideration and conversations with yourself (first and foremost), as well as your manager(s) and mentor(s). Your manager is completing the same tool, gauging what they see as your starting point.

Please complete the tool honestly. Gauging how you honestly see yourself today is a critical starting point for drafting your Plan --- to where you want to see yourself one year from now. Don't worry about trying to be humble, and if there are mindsets or domains with which you aren't familiar or for which you don't know how to respond by all means make a note to this affect in the Comments section. Also, jot down comments in each section about issues or ideas that you wish to discuss with your Institute colleagues, instructors, your manager and/or your mentor.

Please bring your completed Starting Point with you to the June intensive, and if you have any questions or concerns, please contact me –

rebecca@dysartjones.com
905.731.5836

© Copyright 2019 Rebecca Jones

Place a check mark in the column which indicates how frequently you exhibit these knowledge and abilities. The columns are defined as follows:

**1**	**"Always"**	**I *consistently* exhibit this understanding or knowledge**
**2**	**"Frequently"**	**I *almost always* exhibit this understanding or knowledge**
**3**	**"Sometimes"**	**I *sometimes* exhibit this understanding, but I need more experiences and/or deeper knowledge in this area**
**4**	**"Rarely"**	**I have been introduced to this area, but it just *isn't something I do or am familiar with or am comfortable with***
**5 N/A**	**"Unable to comment"**	**I haven't been in a situation that *requires* this type of capability or knowledge**

SELF	**I demonstrate knowledge, confidence and abilities in each of the following areas:**	**1 Always**	**2 Frequently**	**3 Sometimes**	**4 Rarely**	**5 N/A**
	I'm aware of my assumptions, values, principles, what I do well and what is a challenge for me.					
	I actively seeking ways and opportunities – even if they are challenging - that can build my awareness, knowledge, and capabilities.					
	I model and am known for my honesty, integrity, resilience and confidence.					
	I set priorities.					

Comments regarding these areas:

© Copyright 2019 Rebecca Jones

CHANGE	I demonstrate knowledge, confidence and abilities in each of the following areas:	1<br>Always	2<br>Frequently	3<br>Sometimes	4<br>Rarely	5<br>N/A
	I work *in* the Library and *on* the Library.					
	I balance the variables, particularly those in the Library's local, provincial and broader external environment, that drive short and long term success.					
	I identify best practices and norms, and the differences, in the library sector.					
	I discern things that should be changed.					
	I understand the impact of downturns (threats and opportunities), customer behaviour and competitors (existing and emerging).					
	I design strategic options that create flexibility and resiliency.					
	I exercise analytical strength (business intelligence) as well as creativity and intuition (strategic intelligence).					
	I understand the dynamics of complex change, and how to lead it.					

Comments regarding these areas:

© Copyright 2019 Rebecca Jones

Organization	I demonstrate knowledge, confidence and abilities in each of the following areas:	1 Always	2 Frequently	3 Sometimes	4 Rarely	5 N/A
	I know how the Library works, including the structures, procedures and compensation systems,					
	I know how to work the Library.					
	I recognize when and how to use power, influence and persuasion, and who to turn to get things done.					
	I create optimum influence among the greatest number of people.					
	I understand the "nuts and bolts" of the library sector and the factors impacting both the sector and the Library in which I work.					
	I am comfortable with the economics of the library business model, how value is created, how the functions of the business relate to each other, competitive dynamics, customer needs and where the leverage points for improvement reside.					
	I recognize the drivers of operational excellence, the cross-enterprise links to functional areas impacting operations, and affecting efficiency and safety.					

Comments regarding these areas:

© Copyright 2019 Rebecca Jones

RELATIONSHIPS	I demonstrate knowledge, confidence and abilities in each of the following areas:	1<br>Always	2<br>Frequently	3<br>Sometimes	4<br>Rarely	5<br>N/A
	I appreciate individuals and teams, and what it takes to develop and maintain an environment in which people are motivated and that garners their commitment and involvement.					
	I demonstrate values congruent with the organization's, and that convey to employees that they are valued and their contributions matter.					
	I know who can make things happen and the people intelligence to actually make things happen.					

Comments regarding these areas:

© Copyright 2019 Rebecca Jones

CONTEXT	I demonstrate knowledge, confidence and abilities in each of the following areas:	1<br>Always	2<br>Frequently	3<br>Sometimes	4<br>Rarely	5<br>N/A
	I appreciate individuals and teams, and what it takes to develop and maintain an environment in which people are motivated and that garners their commitment and involvement.					
	I demonstrate values congruent with the organization's, and that convey to employees that they are valued and their contributions matter.					
	I know who can make things happen and the people intelligence to actually make things happen.					

© Copyright 2019 Rebecca Jones
