# Supplementary material for: Creating change through leadership development: an overview of the 2019-2021 Canadian Health Libraries Leadership Institute
Source: J Can Health Libr Assoc. 2024 Apr 1;45(1):52–6. doi: 10.29173/jchla29755 (PMC11081116; doi:10.29173/jchla29755)
Supplement: Supplementary file 5 — Supplement Appendix 5 [file JCHLA-45-052-s005.pdf]

Appendix 5: Ending Point

Ending Point

When you began the Institute in May 2019 you and your manager identified your "Starting Point." The Starting Point tool was designed to help you determine your "here" or your starting point for this learning journey. You used it to chart a practical, effective Learning Plan to guide your journey through the past 16 months.

The goal of the Leadership Institute was to provide the opportunity for you to build your capacity to develop a leadership and management approach that integrates five critical components:

1. Self: an understanding of how your perceptions, beliefs and tendencies affect behaviour and workplace performance, and effective strategies for leveraging strengths considering these factors.

2. Context: how to think strategically about the current and emerging environments in which your organization operates and develop action plans to monitor the impact of these and respond continuously.

3. Organization: analyze the functions required for information services in organizations in the health sector, with particular attention to aligning library services to what is critical.

4. Relationships: how to develop and implement strategies to enable multi-directional influence in organizations.

5. Change: how to embrace change as constant and appreciate that change and continuity go hand-in-hand.

Please reflect on your Starting Point, Learning Plan, and the curriculum covered at June 2019's intensive and the virtual sessions. Complete this Ending Point tool, identifying your progress and the Institute's contribution – or where and how the Institute could strengthen its contribution to your growth. Then complete the Institute Evaluation on page

This evaluation will help CHLA/ABSC assess the Institute and help you develop your next learning plan. As an information professional in the healthcare sector, you are on a lifelong journey of learning. Thank you for making the Institute part of that journey.

We look forward to your input. Please send your completed Ending Point to me **by October 10, 2020.**

Rebecca

rebecca@dysartjones.com
905.731.5836

© Copyright 2019 Rebecca Jones

Place an "x" or checkmark in the column indicating how frequently you exhibit this knowledge and these abilities today.

1	"Always"	I ***consistently*** exhibit this understanding or knowledge
2	"Frequently"	I ***almost always*** exhibit this understanding or knowledge
3	"Sometimes"	I ***sometimes*** exhibit this understanding, but I need more experiences and/or deeper knowledge in this area
4	"Rarely"	I have been introduced to this area, but it just ***isn't something I do or am familiar with or am comfortable with***
5	"N/A"	I haven't been in a situation that ***requires*** this type of capability or knowledge

	**I demonstrate knowledge, confidence, and abilities in each of the following areas:**	**1 Always**	**2 Frequently**	**3 Sometimes**	**4 Rarely**	**5 N/A**
**SELF**	a. I'm aware of my assumptions, values, principles, what I do well and what is a challenge for me.					
	b. I actively seek ways and opportunities – even if they are challenging - that can build my awareness, knowledge, and capabilities.					
	c. I model and am known for my honesty, integrity, resilience, and confidence.					
	d. I set priorities.					

Please indicate how the Institute contributed to your growth in SELF (x): **Significantly__** **Somewhat__** **Not at all__**

Please reflect on your learning, the curriculum, and explain your rating:

CHANGE	**I demonstrate knowledge, confidence and abilities in each of the following areas:**	1 Always	2 Frequently	3 Sometimes	4 Rarely	5 N/A
	I work *in* the Library (or unit) and *on* the Library (or unit).[1]					
	I discern things that should be changed.					
	I understand the impact of downturns (threats and opportunities), customer behaviour and competitors (existing and emerging).					
	I design strategic options that create flexibility and resiliency.					
	I exercise analytical strength (business intelligence) as well as creativity and intuition (strategic intelligence).					
	I understand the dynamics of complex change and how to lead it.					

Please indicate how the Institute contributed to your growth in CHANGE (x): **Significantly__** **Somewhat__** **Not at all__**

Please reflect on your learning, the curriculum, and explain your rating:

[1] Working "in": completing assigned tasks, putting out fires; Working "on": building awareness of what's occurring around the organization, where fires may ignite, investing in a fire-prevention mechanism/process

© Copyright 2019 Rebecca Jones

ORGANIZATION	**I demonstrate knowledge, confidence and abilities in each of the following areas:**	1 Always	2 Frequently	3 Sometimes	4 Rarely	5 N/A
	I know how the Library or unit works, including the structures, key services, key processes and compensation systems,					
	I recognize when and how to use power, influence and persuasion, and who to turn to get things done.					
	I create optimum influence among the greatest number of people.					
	I understand the "nuts and bolts" of the Library or information sector and the factors impacting both the sector and the Library or unit in which I work.					
	I am comfortable with the economics of the library/information service business model, how value is created, the lifecycle of the business model, competitive dynamics, customer needs and where the leverage points for improvement reside.					
	I recognize the drivers of operational excellence, the cross-enterprise links to functional areas impacting operations and affecting efficiency and safety.					

Please indicate how the Institute contributed to your growth in ORGANIZATION (x): Significantly__ Somewhat__ Not at all__

Please reflect on your learning, the curriculum, and explain your rating:

© Copyright 2019 Rebecca Jones

RELATIONSHIPS	**I demonstrate knowledge, confidence and abilities in each of the following areas:**	1 Always	2 Frequently	3 Sometimes	4 Rarely	5 N/A
	I appreciate individuals and teams, and what it takes to develop and maintain an environment in which people are motivated and that garners their commitment and involvement.					
	I demonstrate values congruent with the organization's, and that convey to employees that they are valued and their contributions matter.					
	I know who can make things happen and have the 'people intelligence' to make things happen.					
	I know who the Library/unit's key stakeholders and influencers are, including our suppliers, and am adept at building relationships with them to understand their perspectives, goals, and challenges.					

Please indicate how the Institute contributed to your growth in RELATIONSHIPS (x): **Significantly__** **Somewhat__** **Not at all__**

Please reflect on your learning, the curriculum, and explain your rating:

© Copyright 2019 Rebecca Jones

CONTEXT	**I demonstrate knowledge, confidence and abilities in each of the following areas:**	1 Always	2 Frequently	3 Sometimes	4 Rarely	5 N/A
	I know how the parent organization works, including its business model, funding or revenue generation, basic structures, key services, and its position within its sector.					
	I balance the variables, particularly those in the parent organization, local, provincial and broader external environment, that drive short- and long-term success for the Library or unit.					
	I understand the dynamics and developments in the broader environment impacting the parent organization.					
	I avidly observe and consider the developments occurring that impact our suppliers, our users, the departments we serve or collaborate with, and our funders/decision-makers.					
	I identify best practices and norms, and the differences, in the library/information sector.					

Please indicate how the Institute contributed to your growth in CONTEXT (x): Significantly__ Somewhat__ Not at all__

Please reflect on your learning, the curriculum, and explain your rating:

© Copyright 2019 Rebecca Jones

Institute Evaluation

	Strongly Agree	Somewhat	Not at all
1. As a result of the CHLA/ABSC Leader Institute, I have a network of peers and mentors I'm comfortable to turn to for advice and counsel.			
2. As a result of the Institute I have at least two tools or approaches to use as a leader and manager that I did not have – or was not comfortable using – before the Institute.<br>If you respond **Strongly Agree** or **Somewhat,** what are the tools or approaches?			
3. I will recommend the Institute to other information professionals or librarians in the health sector.<br>If you responded **Somewhat** or **Not at all**, please explain:			
4. I, and my sponsoring organization, received a strong return on investment for my participation in the Institute.<br>If you responded **Somewhat** or **Not at all**, please explain:			

5. What parts of the Institute's approach, curriculum or instructors/speakers were particularly meaningful for you?

6. What parts of the Institute's approach, curriculum or instructors/speakers would you advise be changed – and in what way?

We very much appreciate your insights and assessment. Thank you!

© Copyright 2019 Rebecca Jones

Testimonial?

Would you write a testimonial for the Institute? If so, please add it here or send to rebecca@dysartjones.com and siverson@stfx.ca

© Copyright 2019 Rebecca Jones
